# The Effects of Maternal and Postnatal Dietary Methyl Nutrients on Epigenetic Changes that Lead to Non-Communicable Diseases in Adulthood

**DOI:** 10.3390/ijms21093290

**Published:** 2020-05-06

**Authors:** Raniru S. Randunu, Robert F. Bertolo

**Affiliations:** Department of Biochemistry, Memorial University of Newfoundland, St. John’s, NL A1B 3X9, Canada; rbertolo@mun.ca

**Keywords:** developmental origins of adult disease, epigenetics, DNA methylation, methyl nutrients, methionine, choline, betaine, folate, perinatal nutrition, maternal nutrition

## Abstract

The risk for non-communicable diseases in adulthood can be programmed by early nutrition. This programming is mediated by changes in expression of key genes in various metabolic pathways during development, which persist into adulthood. These developmental modifications of genes are due to epigenetic alterations in DNA methylation patterns. Recent studies have demonstrated that DNA methylation can be affected by maternal or early postnatal diets. Because methyl groups for methylation reactions come from methionine cycle nutrients (i.e., methionine, choline, betaine, folate), deficiency or supplementation of these methyl nutrients can directly change epigenetic regulation of genes permanently. Although many studies have described the early programming of adult diseases by maternal and infant nutrition, this review discusses studies that have associated early dietary methyl nutrient manipulation with direct effects on epigenetic patterns that could lead to chronic diseases in adulthood. The maternal supply of methyl nutrients during gestation and lactation can alter epigenetics, but programming effects vary depending on the timing of dietary intervention, the type of methyl nutrient manipulated, and the tissue responsible for the phenotype. Moreover, the postnatal manipulation of methyl nutrients can program epigenetics, but more research is needed on whether this approach can rescue maternally programmed offspring.

## 1. Non-Communicable Diseases, Perinatal Diet and Epigenetics

Non-communicable diseases (NCD) include complicated and overlapping disease entities, which are likely to further develop significantly and become risk factors themselves [1]. NCD, which includes diabetes, cardiovascular disease, obesity, hypertension, and neurodegenerative diseases, has become a leading cause of death in the world. There has been a rapid rise in the incidence of NCD in the past two decades with the incidence projected to continue to rise further. If the primary risk factors for NCD were eliminated, most of the incidence of NCD can be prevented [2]. Thus, international entities have a great interest in not only understanding how to prevent and control these diseases, but also to continue further research to identify novel causes of NCD [3].

The rapid global changes in social and economic aspects have led to changes in dietary and physical activity patterns in the world. The movement of the world towards industrialization with national economies based on trade within a global market has drastically led to deleterious dietary patterns that contribute towards NCD [1]. Thus, the diet has become a critical etiological factor that plays a major role in the development of NCD. However, more recent research has shown that these dietary influences extend quite early in development. Diet during the fetal and neonatal periods can act as a risk factor to develop NCD in later life. This concept, termed “developmental origins of health and disease” (DOHaD), is well described and suggests nutritional (or environmental) perturbations during fetal, infant and childhood stages can permanently program metabolism and predict the development of NCD in adulthood [1,4]. More specifically, research has well established that maternal dietary intake during the perinatal period has a great influence on the later phenotype of the offspring [5]. This direct relationship between early nutrition and later disease was initially observed in the studies of the Dutch winter famine in the Netherlands during 1944, where individuals born to mothers exposed to famine during gestation exhibited increased risks for obesity, cardiovascular disease, insulin resistance, and hypertension in adulthood compared to siblings born during non-famine conditions [6].

Among possible causes of how the maternal diet programs adult chronic diseases, epigenetics has been identified as the leading mechanism [7,8,9]. Epigenetics involves heritable changes in gene expression mediated by extracellular mechanisms that act on DNA, without changing its sequence [10]. For example, cell differentiation and organogenesis are controlled by epigenetic factors through variable regulation of gene expression. The main epigenetic mechanisms involved in the development and differentiation of various cell types are microRNA (noncoding RNAs), covalent histone modification, and DNA methylation. These epigenetic processes involve providing marks on the genome which are responsible for activating or silencing genes leading to a specific phenotype [10]. Thus, they are ultimately responsible for determining phenotypic plasticity [11]. The interaction of nutrients with epigenetics is termed “nutriepigenomics”, where nutrients and their effects on health are mediated by epigenetic modifications. One of the best examples of this concept is the development of a honeybee with identical genomes into a queen or a worker depending on the different diet they feed on: royal jelly or a diet of pollen or nectar, respectively [12]. 

Most of the epigenetic changes that take place during gametogenesis seems to be erased after a certain point of development. It is crucial to understand how epigenetic marks become heritable, as many marks are not completely erased during very early development and gametogenesis [13,14]. Some methylated sites survive and replicate during cell division and the marked DNA is passed along with the histones, leading to persistent influence of these marks on gene expression throughout life [15]. Moreover, many epigenetic changes occur throughout development, pre- and postnatally, and are preserved in mitotic cell division, providing the mechanism by which any epigenetic perturbation during development can persist into adulthood unchanged. Therefore, epigenetic modifications due to early nutritional exposures that affect the offspring’s phenotype is not only taking place via germline modification, but also mitotically in somatic cells with long-term effects on gene expression [16].

Particularly relevant to nutriepigenomics, DNA methylation of promoter regions of genes is a key focus of research. DNA methylation is an epigenetic mechanism where the addition of methyl groups to DNA modifies the function of genes affecting its expression by (usually) inhibiting transcription. Differential methylation of promoter regions of various genes seems to be responsible for the plasticity associated with early programming. Nutrients affecting either S-adenosylmethionine (SAM), the universal methyl donor, or S-adenosylhomocysteine (SAH), an inhibitor of methyltransferases, have the potential to modify methylation, and hence expression, of DNA [17]. The perinatal period, which includes both fetal and postnatal stages, acts as a critical window of development and a period in which epigenetic patterns are known to be modifiable. The epigenetic changes that occur within this critical window of development remain stable beyond the window and into adulthood [18]. Thus, the epigenetic alterations caused by nutritional perturbations during this window are likely to persist into adulthood, potentially predisposing the individuals to an altered metabolism that can lead to NCD in later life [19].

Most recent research has focused on the availability of methyl related nutrients during this critical window of the perinatal period, as these nutrients have a direct effect on availability of methyl groups used for methylation in epigenetics. Much research suggests maternal (i.e., prenatal) dietary methyl nutrients can affect the offspring’s risk for NCD in later life via epigenetic alterations [9]. The early postnatal period is also part of the critical window, during which alterations in diet can also contribute to changes in epigenetics that may persist throughout a lifetime. However, whether dietary methyl nutrients during the early postnatal period can reprogram epigenetic alterations from prenatal perturbations is less understood. Indeed, postnatal epigenetic alterations due to changes in methyl nutrients in the infant diet is an emerging area of research in the field [20,21]. Moreover, defining the critical window of epigenetic susceptibility into postnatal life needs more clarification [22]. It is likely that the critical window time frame depends not only on the genes of interest, but also on the target tissue; for example, late developing organs like the brain likely experience epigenetic alterations into adolescence [23]. 

Since affected traits predisposing the individual to NCD can be inherited via epigenetic changes during the perinatal period, longitudinal studies are required to understand whether early programming of metabolism genes by methyl nutrients are beneficial or detrimental for adult health. As a result, most mechanistic research must be conducted in animal models. However, most animal studies supplement with a combination of methyl nutrients during the prenatal period, expecting to increase methyl metabolism and subsequent influence on epigenetics [24]. Thus, the effects on the epigenome are a result of multiple nutrients [25], complicating the mechanistic explanation and limiting the nutritional relevance to humans since foods are not uniquely rich in methyl nutrients only. Moreover, it is arguably more relevant to the human situation to understand deficiency of methyl nutrients, in addition to excesses, as micronutrient-poor diets are a global concern. Indeed, a typical obesogenic diet comprising of excess ‘empty’ calories might also be considered a micronutrient-poor diet, and the associated deficiency of methyl-related nutrients might explain programming effects of such diets. However, the role of methyl nutrient deficiency in programming of NCD by obesogenic diets is beyond the scope of this review. It is also important to note that the exposure time of methyl nutrient perturbation and the amount of methyl nutrients during the perinatal period will have effects on the phenotypic outcomes. Most studies expose their subjects to methyl nutrients throughout the periconception period, gestation, and lactation. Thus, it is challenging to identify the critical window of the perinatal period that is most susceptible to epigenetic alterations which predisposes the individual to NCD in adult life. Timing of intervention is important to identify not only the deleterious, but also the beneficial epigenetic alterations that could not only predispose, but also prevent development of NCD in adult life. The purpose of this review is to discuss current research findings of methyl related nutrients on epigenetic alterations that lead to NCD development in adulthood. 

## 2. Methyl and Methionine Metabolism Pathways

**Figure 1:**
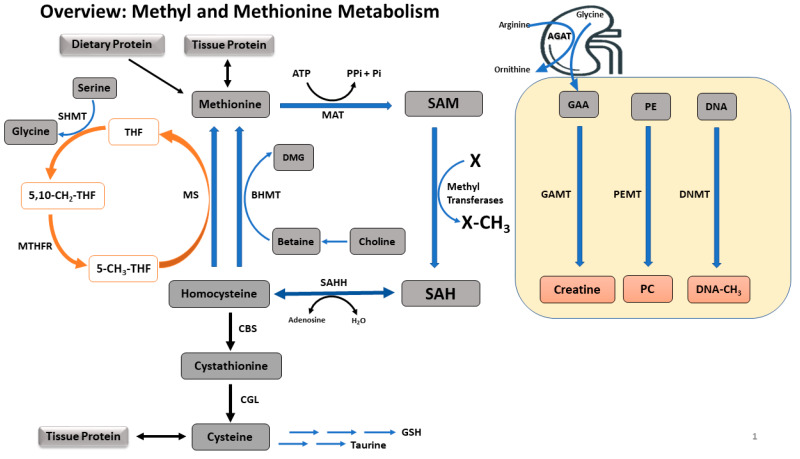
A summary of methyl and methionine metabolism. Methionine is an ubiquitous amino acid acquired via tissue protein breakdown or dietary intake. Methionine is converted to SAM via irreversible adenosylation, which is used for transmethylation. Utilizing methyl groups from SAM, DNA, GAA, PE, glycine, and a number of other transmethylation precursors are transmethylated to methylated DNA, creatine, PC, sarcosine and other products, respectively. These transmethylation reactions are governed by the dietary availability of methionine, choline, betaine, and serine (via folate cycle) which are the primary methyl donors. The common end product of all transmethylation reactions is SAH, which is then converted to homocysteine. Homocysteine is either irreversibly oxidized to cysteine via the transsulfuration pathway or remethylated to methionine via BHMT or MS. Enzymes involved in methyl metabolism are: MAT, methionine adenosyltransferase; GAMT, guanidinoacetate methyltransferase; PEMT, phosphatidylethanolamine methyltransferase; DNMT, DNA methyltransferase; SAHH, S-adenosylhomocystine hydrolase; CBS, cystathionine beta-synthase; CGL, cystathionine gamma-lyase; BHMT, betaine-homocysteine methyltransferase; MS, methionine synthase; SHMT, serine hydroxymethyltransferase; MTHFR, methylenetetrahydrofolate reductase. Metabolite abbreviations: SAM, S-adenosylmethionine; SAH, S-adenosylhomocysteine; GAA, guanidinoacetic acid; PE, phosphatidylethanolamine; PC, phosphatidylcholine; DMG, dimethylglycine; THF, tetrahydrofolate; 5,10-CH3-THF, 5,10-methylenetetrahydrofolate; 5-CH3-THF, 5-methyltetrahydrofolate.

There are three main pathways related to methyl and methionine metabolism: transmethylation, transsulfuration, and remethylation (Figure 1). Methionine is an essential sulfur-containing amino acid and the primary methyl donor for a large number of transmethylation reactions via the methionine cycle. The methionine cycle transfers the terminal methyl group of methionine to form various methylated products and homocysteine (i.e., transmethylation). Homocysteine can be irreversibly oxidized after the transfer of its sulfur atom for cysteine synthesis (i.e., transsulfuration). Or homocysteine can be remethylated to methionine by using methyl groups derived either from betaine via choline, or from methyltetrahydrofolate (THF) generated from the folate cycle (i.e., remethylation). Thus, dietary betaine, choline, and folate are integral in the remethylation pathways through either betaine-homocysteine methyltransferase (BHMT) or through the vitamin B_12_-dependent methionine synthase (MS), and along with dietary methionine, these ‘methyl nutrients’ dictate the dietary availability of methyl groups for transmethylation reactions. Among transmethylation reactions, one of the more important pathways is methylation of DNA via DNA methyltransferases (DNMT) utilizing methyl groups from SAM, the universal methyl donor from methionine. Although the partitioning of methyl groups to DNA methylation via DNMT consumes only ~1% of the total dietary methionine in neonatal piglets [26,27,28], this methylation pathway governs the most critical transmethylation reaction, with respect to long term health of the offspring [7,29]. Early life methylation of cytosine-guanine dinucleotides (CpG) located in the promoter regions of DNA may not utilize quantitatively significant amounts of methyl groups, this repression of gene expression persists throughout the lifetime of an individual, predisposing them to NCD.

## 3. Effects of Maternal Dietary Methyl Related Nutrients on Offspring

Many studies revealing the connection between dietary methyl groups and epigenetics are conducted using maternal dietary interventions during the gestation period only, or during gestation and lactation [25], where it is challenging to isolate the consequences due to prenatal diet or postnatal diet. Studies conducted to evaluate the effects of postnatal methyl nutrients only on either short-term metabolic alterations or long-term adult diseases via epigenetics are rare. Folate, choline, betaine, and methionine are the most important dietary nutrients used in these studies since they play a significant role in methyl metabolism as methyl donors. 

### 3.1. Folate

Among these nutrients, folate has been extensively studied both as a methyl donor and for the effects of supplementation and deficiency in maternal diets on offspring metabolism. Changes in folate metabolism affect epigenetics and early programming via its role in remethylation to methionine to sustain methyl supply during development. Folate determines the flux of one-carbon units towards synthesis or methylation of DNA and RNA and controls methylation regulators via SAM [30]. Folate is a necessary to transfer methyl groups from serine to 5-methylTHF, the remethylation precursor for MS, which transfers methyl groups to homocysteine to synthesize methionine. A cellular deficit of folate leads to decreased synthesis of methionine and the accumulation of homocysteine, resulting in cellular stress [31]. Thus, dietary folate deficiency leads to hyperhomocysteinemia [32].

However, the lack of adequate methionine to maintain epigenetic processes has permanent effects, and has been demonstrated in animal models. For example, a study in rats reported that a gestational folate- and methyl donor-deficient diet led not only to increased plasma homocysteine and hepatic steatosis in dams, but also diminished the body weight, length, folate and SAM/SAH ratio in livers of the fetuses [33]. Notably, low birth weight is a well-identified factor that predisposes to chronic diseases in later life [7]. Reduction in SAM/SAH ratio impairs the capacity of the cell to maintain transmethylation reactions including DNA methylation [33]. As an adaptation to folate deficiency, the methyl donor-deficient group showed activated transcription of folate receptor and Slc19a1 (reduced folate carrier protein) in the fetal liver. The same study also reported that heterochromatin, H4K20me3, which is involved in genome stability, was also significantly decreased in the methyl donor deficient group. The weakening of heterochromatin organization and interference with the expression of genes suggest impaired cell function [33]. The consequences of not maintaining adequate cellular content of SAM, SAH, and SAM/SAH ratio in offspring due to low maternal dietary folate (and vitamin B_12_) levels illustrate the importance of folate in nutritional programming and its link to programming metabolism. In another rodent study, rat dams were fed a diet lacking methyl donors during one month prior to conception, gestation, and lactation, which led to myocardium hypertrophy, with cardiomyocyte enlargement, disturbed mitochondrial alignment, decreased respiratory activity of complex 1 and 11, decreased SAM/SAH ratio, and increased triglyceride concentrations in weaning offspring [34]. Further investigations revealed that impaired fatty acid oxidation and mitochondrial respiration were due to imbalanced methylation and acylation of PGC1-α and SIRT1 genes, which are master regulators in mitochondrial metabolism [34]. The above studies indicate the importance of the role of maternal dietary folate on perinatal development via disturbed epigenetic mechanisms. 

It is relevant that deficiency in folate can lead to chromosomal strand breaks and altered methylation of specific regions of the DNA/ CpG islands, as folate is a critical factor for supplying methyl groups for induction and maintenance of DNA and histone methylation [35]. Indeed, dietary folate deficiency has been identified as a cause for genome-wide hypomethylation [36]. A study in humans found that supplementation of folic acid to pregnant women showed methylation of specific CpG dinucleotides in LINE-1 (long interspersed nucleotide element-1). Methylation of LINE-1 is an assessment of global methylation and was positively associated with offspring birth weight [37]. Although maternal folate supplementation led to global epigenetic changes and improved birth weight, the long-term effects on offspring phenotype was not investigated in the study; however, it is well documented that lower birth weight leads to adult chronic diseases [38]. Thus, these results suggest maternal folate deficiency may predispose offspring to NCD in later life, mediated by low gestational fetal growth via DNA epigenetic modifications.

Collectively, this research shows that folate plays an important role in changing the epigenome, which then governs metabolism. These molecular consequences demonstrate the role of maternal dietary folate and other methyl donors in dysregulation of metabolism via epigenetics, which is heritable. However, the association between the epigenetic effects of maternal folate status and long-term disease phenotype of the offspring needs further research.

### 3.2. Choline

Choline is an important methyl related nutrient that has long been recognized as a substrate crucial for central nervous system development. Choline serves as a metabolic precursor of the major brain phospholipid, PC, and the neurotransmitter, acetylcholine, and is involved in membrane formation and methylation reactions [39,40,41]. The development of the nervous system in utero requires the coordination of neurogenesis and angiogenesis to ensure adequate oxygen supplementation, as well as efficient waste removal from developing neurons [42]. A study in mice reported that maternal dietary choline during pregnancy (D12-17) modulates angiogenesis in developing fetal brain [43]. In contrast, a choline-deficient diet led to the decreased proliferation of endothelial cells in the hippocampus and lower production of blood vessels. Choline deficiency led to higher expression of genes for angiogenic signals (VEGFC and ANGPT2) in endothelial and neuronal progenitor cells, which corresponded with lower DNA methylation of their promoter regions [43]. Indeed, hypomethylation of genes is usually associated with increased gene expression [44]. Thus, maternal dietary intake of choline influences not only neurogenesis [45], but also endothelial cell and blood vessel formation in the fetal brain [43]. These data demonstrate how a maternal diet low in the methyl donor choline leads to a specific physiological phenotype in the fetus.

Supplementation of choline during critical periods of pregnancy can also improve memory performance in adulthood. Choline protects against age-related memory decline, whereas choline deficiency, especially during development, impairs cognitive functions [39]. A rodent study which fed pregnant dams a diet (D11-17) containing varying amounts of choline found that the mRNA and protein expression of histone methyltransferases, G9a and Suv39h1, were directly related to the availability of choline in the liver and frontal cortex [46]. Maternal dietary choline supplementation led to the up-regulation of the H3K9Me2 and H3K27Me3 tags of transcriptionally repressed chromatin, while choline deficiency had improved H3K4Me2, which is associated with the active promoters [46]. These results suggest that maternal choline supply modifies fetal histone methylation and the epigenomic mechanisms contributing to the developmental effects on the offspring. 

Maternal choline intake also leads to changes in global and gene-specific DNA methylation due to alterations in methyl group availability [44,47]. Most maternal dietary choline interventions change expressions of genes in the brains of offspring, including in the frontal cortex and hippocampus, which play a central role in long-term cognitive abilities and memory performance. For example, varied gestational choline supply led to an adaptive epigenomic response in the frontal cortex and fetal liver of rats [48]. Global DNA methylation was increased in choline-deficient rats together with overexpression of DNMT1 mRNA, when choline was supplemented in late gestation (D11-17). Choline deficient offspring had hypomethylated CpG in the *Dnmt1* gene in the liver, and CpG hypermethylation of *Dnmt1* in the cerebral cortex, suggesting there might be a feedback mechanism that counteracts short term choline deficiency. When choline was deficient, the regulatory CpG in *Dnmt1* became hypomethylated, which led to greater expression of DNMT1 and a higher rate of DNA methylation. Conversely, when choline was supplemented to the maternal diet, these CpG were hypermethylated, resulting in the suppression of *Dnmt1* expression [48]. Whether these methylation changes are maintained until adulthood needs further investigation.

The prenatal supply of choline also leads to modifications in the expression of genes that are known to influence learning and memory. For example, choline supplemented rats during pregnancy led to higher expression of the transcription factor Zif268/EGR1 [49,50], which is responsible for memory consolidation and long term memory in the brain cortex and hippocampus. On the other hand, choline deficient rats had higher expression of CaMK11β, which is a multifunctional protein kinase found in neurons of the central nervous system, and GABA_B_ receptor, which is an inhibitory neurotransmitter in the hippocampus [51]. These proteins correlate with cognitive changes and are sensitive to the availability of choline in utero; whether these changes are epigenetically regulated is unknown. In another study, Kennedy et al. [52] showed that maternal choline supplementation during pregnancy (D11-18) in rats prevented long term effects of neonatal iron deficiency on cognitive behaviours. In this study, the deficit in memory recognition due to gestational iron deficiency was attenuated by supplementing choline and the preservation of hippocampal *Bdnf* and *Mbp* expression, which are specific genes for long term memory and regular myelination expression, respectively [52]. These studies suggest gestational choline availability is vital for development of cognitive abilities and memory performance; although not directly measured in these studies, choline’s role in DNA methylation and inherited epigenetics is a likely mechanism for maternal choine supply and programming offspring gene expression.

Maternal dietary choline exposure affects learning and memory in offspring via different mechanisms. Because choline is a key methyl donor, it is likely that DNA methylation and epigenetics is involved in this programming, although most studies in choline have not investigated the methylation pattern of promoter regions of genes responsible for cognition phenotypes. Collectively, maternal nutritional choline status during critical periods of pregnancy affects neurogenesis, angiogenesis, cognitive behaviour, and memory recognition of offspring. Notably, neurogenesis and angiogenesis were inherited via epigenetic modifications, but cognition and memory outcomes are only speculated to be inherited via epigenetics, which would explain the persistence of these traits into adulthood.

### 3.3. Betaine

Choline provides methyl groups via conversion to betaine, the actual methyl donor in remethylation to methionine via BHMT. Betaine is acquired in the body either directly from the diet, or via dietary sources of choline, catalyzed by choline dehydrogenase [53]. The principal physiologic roles of betaine include acting as a methyl donor for BHMT, and as a cellular osmolyte. Through its role as a methyl donor, betaine is known to be critical for human embryonic and fetal development [54]. Unlike the more ubiquitous MS, BHMT is primarily expressed in the liver and kidneys; however BHMT is as important as MS for remethylation and inadequate dietary betaine intake leads to lower remethylation and transmethylation [55], as well as increased plasma homocysteine concentrations and decreased SAM concentrations leading to inefficient hepatic fat metabolism [53]. Clinically, betaine improves vascular risk, protects internal organs, and augments performance [56,57]. 

Similar to choline, previous research on the effects of perinatal dietary betaine supplementation on programming of NCD focused on maternal nutritional interventions. For example, in pigs, maternal dietary betaine can modulate glucose homeostasis in offspring [58]. Neonatal piglets born to sows supplemented with betaine throughout pregnancy had significantly higher serum and hepatic betaine contents, together with significantly greater expression of methionine metabolism enzymes in the liver. Moreover, protein expressions of gluconeogenic enzymes, such as pyruvate carboxylase (PyrC), cytoplasmic phosphoenolpyruvate carboxykinase (PEPCK1), mitochondrional phosphoenolpyruvate carboxykinase (PEPCK2) and fructose-1,6-bisphosphatase (FBP1), were all upregulated along with higher hepatic glycogen content and PEPCK1 enzyme activity. These changes involved epigenetic changes, since maternal betaine supplementation changed the methylation patterns of both histones and CpG islands on the promoters of gluconeogenic genes. The PEPCK1 mRNA was associated with DNA hypermethylation and more enriched repression of histone mark H3K27me3, while PEPCK2 and FBP1 mRNA were associated with DNA hypomethylation and more enriched activation of histone mark H3K4me3. Furthermore, the study also showed that microRNA targeting PyrC and PEPCK1 were suppressed in the liver of piglets from betaine supplemented sows. MicroRNAs are known to participate in post-transcriptional regulation by targeting mRNA degradation and transitional repression. Thus, these data suggest that betaine is involved in the inhibition of microRNA-mediated translation repression in PyrC and PEPCK1 genes, which then explains the upregulation of those genes [58]. This study demonstrates that the DNA methylation machinery involved in glucose homeostasis in pig offspring is a complex gene-dependent process involving several mechanisms, which together work to program metabolism and increase risk for NCD later in offspring.

Not only is glucose homeostasis programmed in offspring by maternal betaine, but the metabolism of galactose has also been shown to be altered by maternal betaine status. A recent study demonstrated that maternal dietary betaine exposure throughout pregnancy led to neonatal piglets with lower serum galactose and a downregulation of expression of galactokinase-1 (GALK1), an enzyme involved in the catabolism of galactose [59]. The downregulation of GALK1 mRNA expression was mediated by the observed *galk1* promoter DNA hypermethylation and enriched repression of histone mark H3K27me3. Furthermore, they reported elevated levels of microRNA processing enzymes, Dicer and Ago2 [59]. Thus, in utero exposure to betaine programs glucose and galactose metabolism in offspring via several epigenetic mechanisms, including DNA methylation of promoters.

Maternal betaine supplementation during pregnancy and lactation seems to program effects not only in F1 generation offspring, but also in the F2 generation [60]. These maternal betaine effects on consecutive generations are epigenetically regulated and are distinct. These effects were induced via the differential modification of insulin-like growth factor (IGF1), which regulates fetal development and postnatal growth. In this rat study, maternal betaine supplementation during pregnancy and lactation affected body weight differentially on F1 and F2 generations. F1 pups had lower weaning weight while F2 pups had higher weaning weight, corresponding to lower and higher serum and liver IGF1 levels, respectively. These IGF1 levels were mediated by a significantly hypermethylated hepatic IGF1 gene promoter in F1 pups, and hypomethylated promoters in F2, demonstrating that perinatal betaine supplementation affects offspring growth via epigenetic mechanisms [60]. Since low birth weight is a well described factor that predicts risk for later diseases, such epigenetic programming of IGF1 by maternal dietary methyl groups may explain some programming outcomes from those models of early origins of adult diseases.

The capacity of betaine to program metabolism in nervous tissue is significant as well. Betaine can influence the pathways of inhibitory neurotransmitter production and recycling [61]. The effects of maternal betaine supplementation seemed to involve programming of not only methylation cycle-related enzymes, but also the DNA content in the hippocampus, suggesting betaine plays an essential role in the brain, similar to its precursor choline. Prenatal betaine supplementation throughout gestation in sows increased the hippocampal DNA content, serum methionine levels, mRNA expression of proliferation-related genes, and mRNA and protein expressions of BHMT, glycine-N-methyltransferase and DNMT1 in piglet hippocampus [62]. Importantly, the hippocampal expressions of IGF2 and IGF receptors were significantly upregulated with maternal betaine supplementation. IGF2 is known to play an essential role in stimulating the proliferation of neuron progenitors via IGF receptor and is critical for hippocampal-dependent memory consolidation and adult hippocampal neurogenesis. Importantly, the effect of betaine was likely epigenetically mediated since the differentially methylated regions (DMR) of IGF2 locus were significantly hypermethylated with betaine exposure [62]. Whether these epigenetic alterations led to a specific phenotype in the offspring as they age is unknown, but programming of cognitive function by methyl groups is well described in other models. 

Maternal betaine supplementation during pregnancy also affects epigenetic regulation of hormonal receptor genes in the brain of offspring. For example, betaine supplementation during gestation in sows enhanced glucocorticoid receptor (GR) expression in the piglet hippocampus including alteration in microRNA expression [63]. The GR receptor in the hippocampus is involved in energy homeostasis and stress sensitivity. Diminished glucocorticoid signaling is associated with an age-related decline in hippocampal function, suggesting changes in GR may persist in the long-term [64]. The effects of betaine on GR expression is likely mediated by methylation given that GR is well known to be epigenetically regulated in the brain and influenced by dietary methyl nutrients [65].

Studies have shown that betaine insufficiency is associated with metabolic syndrome, including lipid disorders and Type 2 diabetes; however, betaine supplementation has also been shown to lead to increased serum total cholesterol and low density lipoprotein (LDL)-cholesterol concentrations [66]. Thus, the effects of supplementation of betaine on lipid metabolism are equivocal and require further investigation. Similarly, maternal dietary betaine supplementation during gestation on lipid metabolism is controversial. Maternal betaine causes complex and differential changes to lipid metabolism of neonatal piglets, with the involvement of epigenetic modifications, including DNA and histone methylation. For example, a study in swine demonstrated that maternal betaine supplementation during pregnancy showed downregulation of sterol regulatory element-binding protein-1c (SREBP1c), a transcription factor involved in cholesterol biosynthesis, 3-hydroxy-3-methyl-glutaryl-coenzyme A reductase (HMGCR), a rate-controlling enzyme in cholesterol biosynthesis, and LDL receptor, a protein that mediates cholesterol clearance, as well as upregulation of cholesterol-27a-hydroxylase (CYP27α1), the rate-limiting enzyme in bile acid synthesis. Furthermore, HMGCR was associated with CpG island hypermethylation and higher repressive histone mark H3K27me3. These results suggest that prenatal betaine supplementation increases hepatic cholesterol in neonatal piglets through epigenetic regulation of genes involved in cholesterol metabolism [58]. However, the same research group also demonstrated that maternal betaine supplementation led to significantly lower plasma triglycerides in piglets with down-regulated hepatic expressions of the lipogenic genes acetyl-CoA carboxylase (ACC), fatty acid synthase (FAS), stearoyl-CoA desaturase (SCD) and SREBP1c. Betaine supplementation also increased serum methionine and liver SAM/SAH ratio and the downregulation of SCD and FAS genes was accompanied by DNA hypermethylation and higher enrichment of the repression histone mark H3K27me3 on the promoter [67]. Taken together, although maternal betaine supplementation in pregnancy reduces plasma triglyceride levels and lipogenic gene expression, it also increases plasma cholesterol levels; whether these changes should be interpreted in context of neonatal development rather than as cardiovascular risk factors needs to be considered. Regardless, it is clear that betaine affects the expression of genes regulating lipid metabolism and this is mediated through epigenetic mechanisms.

There are very few studies in humans on the effects of betaine on epigenetically mediated outcomes. However, in the maternal nutrition and offspring epigenome cohort study, maternal folate and betaine supplementation epigenetically altered genes related to growth (IGF2), metabolism (retinoid X receptor-α), and appetite (leptin) in offspring [68]. Moreover, these remethylation supplements were associated with lower leptin gene methylation in infants at birth and at six months of age. The longer-term persistence of these epigenetic changes are unknown, but likely also depends on postnatal nutrition of the infants.

Most of the above summarized research investigated the metabolic and epigenetic effects of maternal betaine on offspring in the neonatal stage of development, or the immediate phenotype of offspring. Studies that focused on perinatal dietary metabolic and epigenetic effects on the offspring in the long term are scarce. Moreover, many studies provided dietary methyl nutrients during most of gestation and throughout lactation, thus the effects can only be identified as perinatal effects, and not specifically as prenatal or postnatal effects. Nevertheless, it is clear that maternal betaine exposure can influence the methylation status of offspring in utero, or during lactation, and these effects can be permanent via epigenetic mechanisms.

### 3.4. Methionine

Several research studies have used methionine as a methyl donor to investigate perinatal methyl supply on metabolism and epigenetics of the offspring until weaning. Methionine is an essential amino acid that acts as the universal donor of methyl groups via SAM synthesis and transmethylation. So, it is not surprising that changes to methionine exposure during early development would lead to programming of various metabolic outcomes via epigenetic mechanisms.

Several key studies on the effects of dietary methionine in the perinatal period used ruminants as the animal model. Because ruminants can synthesize their vitamin and amino acid needs in the rumen, there are obvious limitations to extrapolating nutritional conclusions from these models to human diets. On the other hand, the metabolic effects of methyl nutrients on epigenetics are still relevant in ruminants. Feeding rumen-protected methionine to pregnant cows during the perinatal period led to higher concentration of methionine in maternal plasma, but not in colostrum [69]. However, a higher abundance of enzymes related to methionine metabolism (i.e., BHMT, SAHH, CBS, MAT, glutamate cysteine ligase, glutathione reductase, cysteine-sulfinate decarboxylase) was observed in the livers of calves from methionine supplemented cows. Interestingly, maternal methionine particularly altered the postnatal use of homocysteine for taurine and glutathione synthesis, suggesting the offspring from cows supplemented with methionine may have better control of metabolic-related stress due to a higher amount of taurine and glutathione [69]. In another study, cows supplemented with methionine during the prepartum period had lower plasma concentration of haptoglobin, an inflammation-related biomarker [70]. The gene expressions of inflammatory biomarkers CASP8, MPO, ZBP1, and TNF were lower at birth in the calves, and corresponded with an upregulation of microRNA (i.e., MIR125b, MIR146a, MIR155) [70], which are involved in cell adhesion, chemotaxis, oxidative stress, and receptor signaling of proinflammatory signals [71]. Although not directly measured, the authors suggested that maternal methionine supplementation led to programming of inflammatory functions of offspring via epigenetic mechanisms [70]. All of these findings demonstrate that the perinatal supply of methionine can regulate the development of immune functions in the offspring. 

The same research group also showed effects of maternal methionine on other metabolic outcomes in calves [72]. Dietary rumen-protected methionine during late pregnancy affected 23 blood biomarkers and 24 liver transcriptomes in neonatal calves and by 50 days after birth, methionine supplemented calves had higher plasma insulin and lower glucose levels, indicating greater systemic insulin sensitivity. Furthermore, higher expression of FBP1 and PEPCK1 suggested that calves that received maternal methionine underwent a faster maturation of gluconeogenesis and fatty acid oxidation in the liver, allowing them to more rapidly adapt to extrauterine life. Methionine supplemented calves also showed a lower concentration of reactive oxygen metabolites at 14 d of age, indicating a lesser degree of stress [72]. Maternal dietary methyl nutrient intake can program key metabolic pathways in offspring at weaning, which suggests epigenetic mechanisms are at play; however, the direct effects of maternal methionine on epigenetic markers on specific genes in offspring need to be demonstrated.

Several studies have also demonstrated that maternal supply of methionine can program phenotypes in offspring that persist into adulthood. In a study in sheep, restriction of dietary methionine and vitamin B_12_ during the periconceptional phase led to higher body weight, altered immune response to antigenic challenge, insulin resistance, and elevated blood pressure in adulthood [73]. These changes were coupled with altered methylation status of 4% of 1400 CpG islands in the fetal liver, and half of the loci were specific to males, demonstrating nutritional programming of sex-specific phenotypes via widespread epigenetic changes [73]. In protein-restricted rats, offspring from dams supplemented methyl donors (methionine, folic acid, betaine, choline) had reduced adipose leptin secretion because of higher methylation of the leptin gene promoter [74]. Demonstrating persistent programming, the effects on offspring in this study were observed short term (at birth, weaning) as well as long term (week 23). These effects led to impaired postnatal growth in offspring, but not food intake, further indicating that maternal methyl donor supplementation programs energy homeostasis mechanisms via epigenetics, and these effects persist into adulthood.

From another perspective, dietary methyl groups can also prevent the adverse effects of a poor maternal diet on the offspring. A study in mice showed that dietary supplementation of methionine throughout gestation and lactation prevented the adverse effects (i.e., obesity, glucose intolerance, insulin resistance) of maternal high fat diet on offspring [75]. Expression and DNA methylation profiles of obesogenic genes (i.e., PPARγ, FAS, leptin, adiponectin) in visceral fat were decreased in offspring from dams fed high fat plus methionine diets, compared to those from dams fed diets high only in fat. Accordingly, the individual CpG island methylation levels in the promoter regions of these genes were significantly enhanced in offspring exposed to high maternal dietary methionine which persisted at ages 8, 12, and 16 weeks, demonstrating the programming of epigenetic profile [75]. Thus, maternal dietary methionine can reverse adverse programming effects from a poor maternal diet, although it would be more relevant if a postnatal diet can rescue the offspring that has been programmed by maternal diet. Such an intervention would need to occur within the window of programming susceptibility, but would provide a potent strategy to overcome the deleterious maternal dietary exposures on offspring metabolism.

## 4. Effects of Postnatal Dietary Methyl Related Nutrients on Adult Phenotype

Much of the aforementioned research demonstrated that perinatal methyl donor supplementation can epigenetically program metabolism in offspring; however, fewer studies clearly separate prenatal and postnatal dietary interventions and their effects on epigenetic alterations leading to adult phenotypes. Much evidence has accumulated that the ‘window’ of susceptibility to nutritional programming of chronic disease spans pre- and postnatal phases, but less information compares the two stages with respect to phenotype. The lactation/postnatal period is likely as important as the pregnancy/prenatal period since many developmental processes are completed within the immediate postnatal period. Even more complicated for translation to humans, different species are at different developmental stages at birth. For example, rodents are altricial species and much more developmental maturation occurs postnatally compared to precocial species such as pigs and humans. Moreover, different organ systems mature at different developmental stages, so some disease outcomes are programmed prenatally (i.e., hypertension, Type 2 diabetes) and others postnatally (i.e., cognition, obesity) [23]. 

In most mammals, milk is the sole nutrient source for neonates. Because maternal nutrition and metabolism can shape milk characteristics, maternal nutrition during lactation has a key role in programming the metabolism of suckling offspring. However, infant formulas often replace mammary milk in humans, and commercial infant formulas have a wide range of methyl nutrient content, thereby potentially altering methyl-induced epigenetic programming [21]. To some extent, the metabolic effects that are inherited due to maternal diet during gestation is less controllable than postnatally, and programming effects are already defined for an individual at birth. However, there are more opportunities to modify the postnatal diet to restore the deleterious epigenetic effects of the maternal diet, via use of modified infant formulas and carefully designed weaning diets. Some studies have tested this hypothesis in rodent models. For example, Cordero and colleagues [25,76] showed that methyl donor supplementation (i.e., choline, betaine, folic acid, vitamin B_12_) during lactation to dams fed an obesogenic diet reduced liver fat accumulation in the offspring at 20 weeks of age, demonstrating the hepatoprotective effect of postnatal methyl nutrients. Postnatal methyl supplementation also corresponded with a higher hepatic leptin receptor expression along with altered DNMT expression, suggesting that epigenetic processes are involved in determining the liver transcriptome profile and metabolism [25,76]. This rescue effect by postnatal folate was also demonstrated in study in rats [77]. Interestingly, the obesogenic phenotype and impaired glucose response in offspring from dams fed high folate diets was reversed by feeding pups a high folate postnatal diet. Moreover, these effects were mediated by changes in the methylation of the promoter of pro-opiomelanocortin (POMC), which is involved in appetite suppression, by postnatal folate [77]. This post-weaning epigenetic plasticity allows a mechanism to reverse in utero programming by maternal diets. These studies demonstrate that postnatal methyl donors may be useful in reversing epigenetic programming to reduce the incidence of obesity and non-alcoholic fatty liver disease, key components of metabolic syndrome. More studies are needed to understand the differential effects of postnatal diets on epigenetics, as the potential for rescuing maternally programmed offspring is significant.

## 5. Conclusions

When considering all the above studies together, it is apparent that the timing of nutrient perturbation contributes to the epigenetically programmed alterations in physiology and metabolism observed in offspring. The critical window of susceptibility to epigenetic programming occurs during the rapid growth and maturation phase of the offspring during the perinatal period, which includes gestation and lactation. In turn, the disease outcome that is programmed depends on the critical window of susceptibility of specific genes governing specific functions in individual tissues. For example, kidneys are more sensitive to programming during the phase of nephrogenesis that occurs during the prenatal period [78]. Whereas, the critical period of development for the brain extends well into childhood because of its continuous postnatal development of neural pathways [79]. Therefore, a more extended period of dietary exposure may have a broader impact on functional outcomes of many developing organs and systems. Apart from the timing, the type of methyl donor and the amount of supplementation also contribute to the manifestation of the observed metabolic changes, mediated by the epigenetic alterations. These factors need to be considered in methyl nutrient dietary interventions in order to elucidate the complex phenotypic outcomes due to specific epigenetic changes. Moreover, the window of susceptibility of these alterations needs to be known, since exposure to methyl nutrients is very dynamic from maternal nutrition during stages of pregnancy, to infant feeding, and to diets in childhood and adolescence. 

Although there is substantial evidence that dietary methyl donor status during the perinatal period can lead to the programming of metabolism leading to NCD, more studies are needed that investigate how specific nutrients directly affect epigenetic changes that lead to the disease phenotype. Such knowledge would help develop postnatal dietary strategies that might reverse the epigenetic effects caused by maternal nutrition. In this way, it is important to advise expectant mothers to focus on a healthy, balanced diet during pregnancy, and to emphasize the importance of breastfeeding. Until we have more information, it is critical to not deviate from ‘normal’ healthy nutrition and to avoid extreme exposures to methyl nutrients in order to prevent programming of the metabolism that could lead to NCD in adulthood.

## Abbreviations:

ACC	Acetyl-CoA carboxylase
BHMT	Betaine-homocysteine methyltransferase
CBS	Cystathionine beta-synthase
5,10-CH_3_-THF	5,10-methylenetetrahydrofolate
5-CH_3_-THF	5-methyltetrahydrofolate
CGL	Cystathionine gamma-lyase
CpG	Cytosine-guanine phosphodiester dinucleotide
CYP27α1	Cholesterol-27a-hydroxylase
DOHaD	Developmental origins of health and disease
DMG	Dimethylglycine
DMR	Differentially methylated region
DNA	Deoxyribonucleic acid
DNMT	DNA methyltransferase
FAS	Fatty acid synthase
FBP	Fructose-1, 6-bisphosphatase
GAA	Guanidinoacetic acid
GABA_B_	Gamma-aminobutyric acid
GALK1	Galactokinase-1
GAMT	Guanidinoacetate methyltransferase
GR	Glucocorticoid receptor
HMGCR	3-Hydroxy-3-methyl-glutaryl-coenzyme A reductase
IGF	Insulin-like growth factor
LDL	Low density lipoprotein
LINE-1	Long interspersed nucleotide element-1
MAT	Methionine adenosyltransferase
mRNA	Messenger RNA
MS	Methionine synthase
MTHFR	Methylenetetrahydrofolate reductase
NCD	Non-communicable diseases
PC	Phosphatidylcholine
PE	Phosphatidylethanolamine
PEMT	Phosphatidylethanolamine methyltransferase
PEPCK	Phosphoenolpyruvate carboxykinase
PPARγ	Peroxisome proliferator-activated receptor gamma
PyrC	Pyruvate carboxylase
RNA	Ribonucleic acid
SAH	S-adenosylhomocysteine
SAM	S-adenosylmethionine
SAHH	S-adenosylhomocystine hydrolase
SCD	Stearoyl-CoA desaturase
SHMT	Serine hydroxymethyltransferase
SREBP1c	Sterol regulatory element-binding protein-1c
THF	Tetrahydrofolate
